# Ossification Vesicles with Calcium Phosphate in the Eyes of the Insect *Copium teucrii* (Hemiptera: Tingidae)

**DOI:** 10.1100/tsw.2011.9

**Published:** 2011-01-18

**Authors:** Javier Garcia-Guinea, Alberto Jorge, Laura Tormo, Marta Furio, Elena Crespo-Feo, Virgilio Correcher, Pedro Prado-Herrero, Ana C. Soria, Jesus Sanz, Jose L. Nieves-Aldrey

**Affiliations:** ^1^ Museo Nacional de Ciencias Naturales (MNCN, CSIC), Madrid, Spain; ^2^ CIEMAT, Dosimetria de Radiaciones, Madrid, Spain; ^3^ Instituto de Quimica Organica General (CSIC), Madrid, Spain

**Keywords:** gall-inducer, insect, eye, Copium, Teucrium, hydroxyapatite, ossification, Raman, luminescence, GC-MS

## Abstract

Arthropod eyes are built of repeating units named ommatidia. Each single ommatidium unit contains a cluster of photoreceptor cells surrounded by support cells and pigment cells. The insect *Copium* eye ommatidia include additional calcium-phosphate deposits, not described in insects to date, which can be examined today using a combined set of modern microscopy and spectroscopy techniques. *Teucrium gnaphalodes* L'Her plants, growing in central Spain, develop galls induced by *Copium* insects. A survey of *C. teucrii* adult specimens resulted in surprising environmental scanning electron microscopy (ESEM) images, showing that their bright red eyes contain a calcium-phosphate mineralization. A complete survey of *Copium* eye specimens was performed by ESEM using energy-dispersive spectroscopy, backscattered electron detector and cathodoluminescence (CL) probes, field emission scanning electron microscopy, micro-Raman spectroscopy, and confocal laser scanning microscopy in order to learn ommatidia features, such as chemical composition, molecular structure, cell membrane, and internal ommatidium eye fluids and calcium-phosphate distribution deposits. The CL panchromatic images distinguish between the calcium-phosphate ommatidium and calcium-phosphate setae, which are more apatite rich. They show Raman bands attributable to bone tissue apatite biomaterials, such as bone, collagen, lipids, and blood, i.e., peptides, amide-S, amide-II, amide-III, and cytochrome P-450scc. The chemical composition of both galls and leaves of *T. gnaphalodes* was determined by gas chromatography – mass spectrometry (GC-MS) of their extracts. The spectrometric and microscopic images reveal that the calcium-phosphate mineralization is formed and constrained to *Copium* ommatidia, which are both matrix vesicles generating mixtures of apatite collagen and operational compound eyes of the insect.

## INTRODUCTION

In vertebrate species, during endochondral ossification, extracellular matrix vesicles sized ~100 μm in diameter generate calcium phosphates, such as octacalcium phosphate (OCP), amorphous calcium phosphate (ACP), hydroxyapatite (HAP), or dicalcium phosphate dehydrate (DCPD), nucleated from organic phosphatases, plus calcium-binding molecules in cartilage, bone, and predentin. Similar ossification processes are scarcely described in insects, e.g., calcium phosphate in the exoskeleton of larval *Exeretonevra angustifrons*[1]. In the insects case, a diversity of metals, including zinc, manganese, and iron, are found concentrated within mandibles, mouth hooks, claws, and ovipositors[2,3,4]. The presence of metals modifies the mechanical properties of the cuticle performing different biological functions not yet completely understood. Entomologists recurrently using the backscattering detector and the chemical probes under the electron microscopes of our working center, i.e., Museo Nacional de Ciencias Naturales (MNCN, Madrid, Spain) to identify metals, observed a calcium-phosphate mineralization in the red eyes of *Copium teucrii*. These insects induce galls on *Teucrium gnaphalodes* plants (Fig. 1a), which grow in the northern area of Madrid called Torrelaguna. Galls occur in *Teucrium* flowers, whose petals swell and thicken at the bottom, and develop from April to June depending on the localities and microclimates. Inside the galls, we find both larvae and adult specimens, which get outside by drilling a hole in the gall wall (Fig. 1b). The color of insect eyes is determined largely by the nature of the screening pigments, e.g., ommochromes, derived from the amino acid tryptophan, and pteridins derived from guanine nucleotids[5]. It has been found that calcium is a structural component of ommochrome pigment granules of the eyes in species of insects groups, such as mosquitoes, moths, and dragonflies[6]. Eye pigments have been studied in at least two hemipteran species related to the target species of this work, e.g., the black (wild) and red (mutant) eyes present in *Triatoma infestans* (*Reduviidae*)[7] and *Reduviid hemipteran* and *Rhodnius prolixus* also with eye pigments[8]. However, no data have been reported on the composition of eye pigments of the gall-inducer *Copium* species. *Teucrium* plants usually grow in Mediterranean climate areas on basic substrates in low and middle levels. In our case, the geological stratigraphic columns are made of beds of limestone, marl, and gypsum materials occupying the Tertiary sedimentary basin next to the town of Torrelaguna. Among the more than 100,000 species of many insect groups and mites that are capable of inducing galls on plants, the order Hemiptera is represented by just only a dozen of species of the genera *Copium* and *Paracopium* (Tingidae)[9,10]. The insects of the Palaearctic genus *Copium* induce galls on plants of species of *Teucrium* (Lamiaceae). The most common is *C. teucrii* (Host), which induces galls on flowers of *T. polium* and *T. gnaphalodes*. *Copium* species were taxonomically revised in 1979 by Pericart[11], while the interaction between *C. teucrii* and its host plant *T. polium*, including observations on the gall-inducer life cycle and the morphological, anatomical, and hormonal changes in the plant, were performed in a Sinai desert ecosystem[12]. From a chemical point of view, several neoclerodane diterpenoids and flavonoids[13,14,15] have been found in *T. gnaphalodes*, but no determination of its general chemical composition was published. Gall formation in *Teucrium* has been scarcely studied[16,17]. The intense bright red color of the *Copium* eyes, coupled with the calcium-phosphate composition detected in the energy-dispersive spectroscopy – environmental scanning electron microscopy (EDS-ESEM) chemical analyses, suggested to us broad knowledge on this mineralization. After 4 decades of Raman spectroscopy studies of calcified tissues, starting in the 1970s[18], the Raman molecular technique has been used to differentiate collagen, gelatin, and elastin materials for biopolymer uses[19]; to separate normal and diseased human breast tissues[20]; to discriminate surrounding tissue of basal cell carcinoma[21]; or to study ultrastructural changes accompanying the mechanical deformation of bone tissue[22]. For the assignment of Raman bands and peaks ($\Delta cm^{-1}$), we followed the table of Penel et al.[23] for bone tissue apatite biomaterials, such as bone, collagen, lipids, and blood. Additionally, we also took into account other Raman studies on peptide bonds with amide-S[24], the heme moiety of cytochrome P-450scc[25], and a quantitative methodology for determining protein secondary structure[26] for the Raman assignations of our insect specimen. Here we study the *Copium* eyes case as membrane-bound matrix vesicles calcium-phosphate–bearing and cytochrome phases by ESEM using

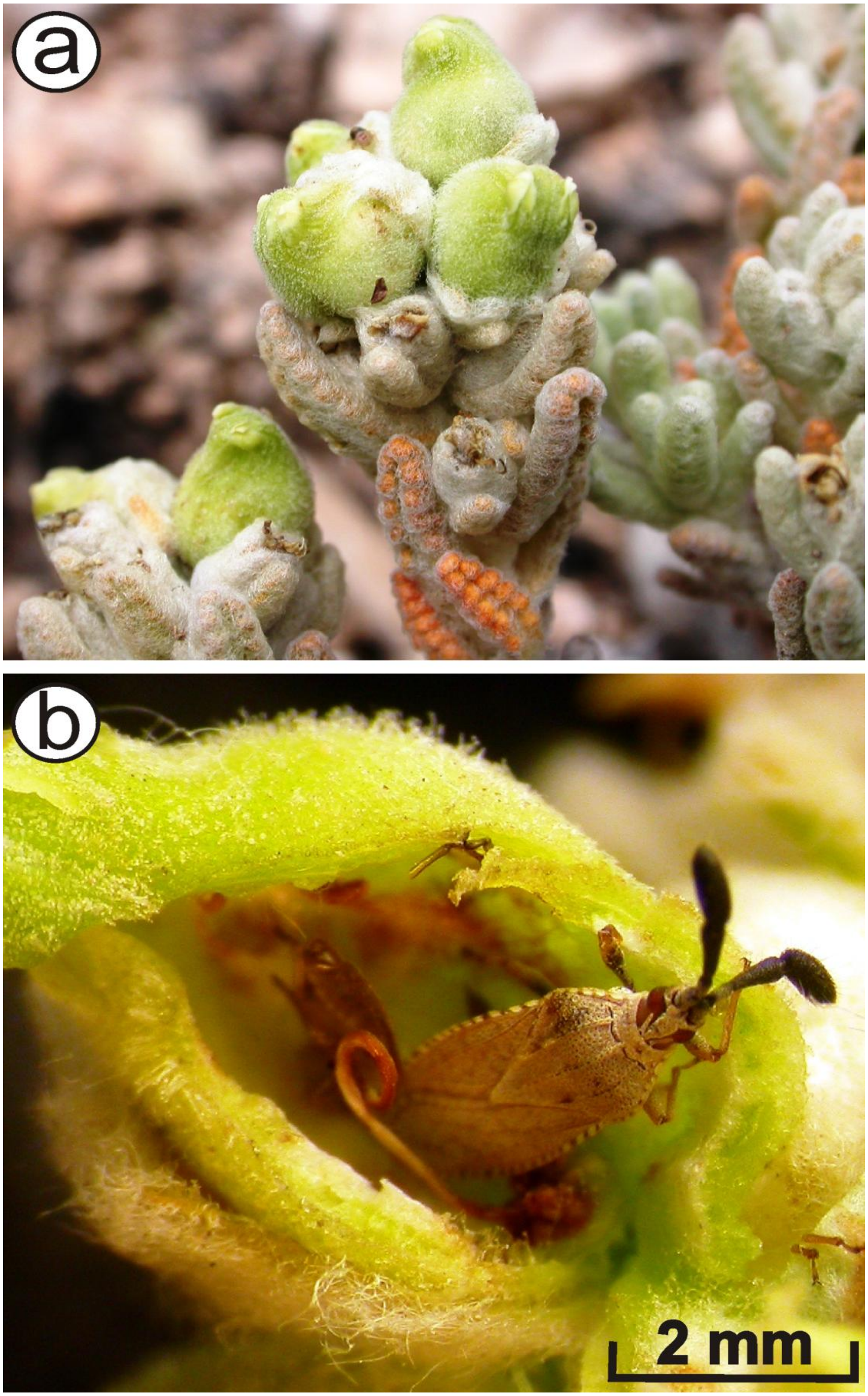

**FIGURE 1.** (a) Vegetable galls induced in *Teucrium* flowers by the *Copium* insect (Torrelaguna, Northern Madrid, Spain); (b) adult *Copium* insects are totally formed in the interior of galls and get outside by drilling a hole in the gall wall.

EDS, backscattered electron detector (BSED) and cathodoluminescence (CL) probes; field emission scanning electron microscopy (FESEM), micro-Raman spectroscopy (RAMAN), and confocal laser scanning microscopy (CLSM**).** The organic compounds in gall and leaves were also studied by gas chromatography – mass spectrometry (GC-MS).

## SAMPLES AND EXPERIMENTAL METHODS

Nymphs and adults of *C. teucrii* insects were collected during a few weeks in the late spring months in which adult specimens exit the plant galls (Fig. 1b). The insects studied here were identified following the revision of Pericart[11]. The scarce amount of sample for analyses, limited to insect eyes, suggested to us to perform microscopy studies using several coupled analytical probes. The ESEM XL30 microscope of the FEI Company settled in our working center (MNCN, Madrid) is a low-vacuum ESEM that enables high-resolution inspection and chemical analysis of nonconductive specimens. The ESEM microscope in low vacuum mode admits hydrated samples to be studied in their original state with the large field detector (LFD), since it is close to the sample in order to avoid electron losses. This ESEM can also work at high vacuum conditions with samples covered with sputtered gold, providing a better resolution in electronic images and more accurate chemical analyses by EDS. The ESEM resolution at high vacuum was 3.0 nm at 30 kV (SE), 10 nm at 3 kV (SE), and 4.0 nm at 30 kV (BSE). While operating at low vacuum, it was 3.0 nm at 30 kV (SE), 4.0 nm at 30 kV (BSE), and <12 nm at 3 kV (SE). The accelerating voltage was 200 V to 30 kV and the probe current up to 2 μA was continuously adjustable. The ESEM detectors are as follows: the LFD, Everhardt-Thornley or high-vacuum secondary electrons detector (SED), the IR-CCD camera, a solid-state BSE detector, and a new gaseous analytical electron detector (GAD). Precalibration tests of ESEM-EDS chemical measurements were previously performed on bond-HAP and hydrothermal fluorapatite standards to improve the ZAF correction procedure. A series of aliquot samples was tested by both chemical probes, wavelength-dispersive spectroscopy (WDS), and EDS, placed in the ESEM Quanta 200 also in our MNCN laboratories. The oxygen, phosphorous, and calcium peaks for each point of analysis was determined one by one, by a peak-seek routine on the WDS detector. The EDS spectra and the oxygen X-rays obtained by WDS were collected for each sample. The total oxygen intensity was transferred to the EDS unit for data reduction using a multiple least-squares fitting program to compare the spectra against the standards. The organic oxygen concentrations were also calculated from the observed oxygen intensities using the "ZAF" program to correct atomic number, absorption, and fluorescence effects in the apatite matrix. In addition, this SEM has a new coupled MONOCL3 Gatan probe to record CL spectra and panchromatic and monochromatic plots with a PA-3 photomultiplier attached to the ESEM. The photomultiplier tube covers a spectral range of 185–850 nm and is more sensitive in the blue parts of the spectrum. A retractable parabolic diamond mirror and a photomultiplier tube were used to collect and amplify luminescence. The sample was positioned 16.2 mm beneath the bottom of the CL mirror assembly. The excitation for CL measurements was provided at 25-kV electron beam. The hyperspectral calcium-phosphate and protein distribution was also explored with a Thermo Fisher DXR Raman microscope, which has a point-and-shoot Raman capability of 1-μm spatial resolution using a laser source at 532 nm. For this case of organic materials, we prefer to use a second 780-nm excitation laser. The FESEM microscopy was performed with a JEOL JSM-6330F microscope operated at 3 kV and equipped with an Oxford energy-dispersive microanalysis system and Inca software. *Copium* were previously treated by progressive dehydration with acetone and coated with sputtered graphite for electron-conducting purposes. For the CLSM analyses, the confocal microscope used was a Leica TCS SPE-DM 5500 Q V-Vis instrument equipped with three solid-state lasers operating at 488, 532, and 635 nm; an HC PLAN 10× eyepiece; an HCX PL FLUOTAR 10×/0.30, 40×/0.75 dry objectives; an HC PL APO 20×/0.70 dry objective; and the Leica Application Suite Advanced Fluorescence software. Fluorescence emission was collected from approximately 5 nm above the excitation wavelength up to 750 nm. Laser power for acquisition was set by viewing the fluorescence emission and increasing the power up to the rate of increase when fluorescence appeared to slow. The photomultiplier gain for acquisition was then set by viewing the image and increasing the gain until signal overload was detected, at which point the gain was backed off slightly. 512 × 512 pixels were acquired for each Z-step; a zoom setting of 1.5 in sequential capture. Data acquisition parameters were chosen that allowed collection of serviceable images within a few minutes. Images shown have not been enhanced.

Concerning the chemical analyses of the galls and leaves of the insect host, the *T. gnaphalodes* plants, the sample fractionation previous to GC-MS analysis was performed using solvent extraction, taking into account the small amount of plant gall available and our interest in analyzing compounds of a broad volatility range. Internal standard (*n*-eicosane) was added to dried galls and leaves, which then were extracted with methylene chloride using sonication. Analyses of the extracts were carried out in an Agilent 6890A gas chromatograph coupled to a mass detector Agilent 5973, using a fused silica capillary column (30 m × 0.25 mm × 0.25 μm) coated with 5% phenyl methylpolysiloxane. 1 μL was injected in the splitless mode. The column was programmed from 60ºC (1 min) to 290ºC, using 6ºC/min as programming rate. Retention and mass spectral data were compared with those of data libraries or with references for qualitative analysis. Quantification used the total ion current (TIC) GC-MS areas, supposing the same response factor for all the compounds determined.

## RESULTS AND DISCUSSION

As all the Hemiptera, the *Copium* species exhibits an incomplete metamorphosis, being subjected to a gradual transformation in which the larva resembles the adult, but differs mainly in being of smaller size, in having shorter wings, and in not being sexually mature. Inside the gall, *Copium* has larval development shield, nutrition, and protection against weather and predators. They differ from other gall structures built in nurturing and protecting plants from insects, such as curls or mines in the leaves. This formation process involves active growth and differentiation of plant tissues. *Copium* is able to “induce” the gall formation in the *Teucrium* plant, producing new nutritious and protective tissues for its exclusive benefit. Tables 1a and 1b list the qualitative and quantitative composition of *Teucrium* gall and leaf samples analyzed by GC-MS. The identified compounds mainly belong to two chemical types: terpenoids (Table 1a) and *n*-alkanes (Table 1b). Most identified terpenoids have a sesquiterpene structure (15 carbon atoms); their molecular weights being in the 200–240 a.m.u. range. Monoterpenoids (10 carbon atoms) and some of their acetates are also present. Functionalized terpenoids with molecular weights above 270 a.m.u. have also been detected and in some cases identified (montanin-A,[13]). Twenty other unidentified terpenoids with molecular weights in the 176–234 a.m.u. range were also present in *Teucrium* leaves in concentrations lower than 0.1 mg $g^{-1}$; only a few of these components were also detected in gall samples. *n*-Alkanes having between 23 and 35 carbon atoms have also been determined. Several *n*-alkanes having an odd carbon number (from *n*-heptacosane to *n*-tritiacontane) appear as major constituents both in galls and leaves, while series with an even carbon number are present in lower concentrations (Table 1b).

These detected compounds are secondary metabolites, and their role seems to be more associated with the protection of the insect rather than, as for primary metabolites, with their growth and reproduction processes. In this line, mono- and sesquiterpenes are naturally present in plants and insects as semiochemicals, (e.g., defensive agents or pheromones) and could play related roles in *Teucrium* plants, helping in the chemical protection of *Copium* larvae. High-molecular-weight *n*-alkanes, the main components found in galls, could afford an additional physical protection as constituents of epicuticular waxes, although in some cases the role of *n*-alkanes as pheromones has also been reported. The remarkable red color of the *Copium* eyes, its calcium-phosphate biomineralization, its condition of gall-inducer in *Teucrium*, and the calcium-sulfate substratum are different features necessarily linked by biochemical reactions. The surrounding ground of gypsum ($CaSO_4 \cdot 2H_2O$) is a great provider of calcium for biogenic apatite and sulfur for the cystine compounds. We focus the analytical work on *Copium* adult specimens by the initial surprising backscattering ESEM images that highlight their natural bright red eyes on light color, pointing explicitly to a calcium-phosphate mineralization, since “heavy” calcium and phosphorous atoms depict white BSED halftones in comparison with the carbon BSED dark gray halftones

## ORGANIC COMPOUNDS

***Table 1a.*** - Concentration (mg $g^{-1}$ of sample) of organic compounds present in samples of galls and leaves of *Teucrium gnaphalodes* determined by GC-MS analysis of their $CH_2Cl_2$ extracts.

| COMPOUND | Leaves | Galls |
|---|---|---|
| α-pinene | 0.377 | 0.0045 |
| β-pinene | 0.791 | 0.0079 |
| limonene | 0.088 | 0.0007 |
| 4-methyl-4-vinylbutyrolactone | - | 0.0015 |
| *cis*- + *trans*-linalyl oxide | - | 0.0142 |
| β-pinone | 0.047 | 0.0151 |
| myrtenal | 0.139 | 0.0192 |
| verbenone | 0.032 | - |
| carvone | 0.005 | - |
| linalyl acetate | 0.270 | - |
| bornyl acetate | 0.022 | 0.0024 |
| pinocarveyl acetate | 0.006 | - |
| myrtenyl acetate | 0.009 | - |
| cyclosativene | 0.224 | - |
| copaene | 0.207 | - |
| β-bourbonene | 0.030 | - |
| β-cubebene | 0.044 | - |
| β-caryophyllene | 0.316 | 0.0065 |
| β-copaene | 0.014 | - |
| α-guaiene | 0.005 | - |
| α-humulene | 0.094 | - |
| allo-aromadendrene | 0.019 | - |
| germacrene D | 0.107 | - |
| β-selinene | 0.089 | - |
| δ-cadinene | 0.040 | - |
| α-calacorene | 0.014 | 0.0030 |
| caryophyllene oxide | 0.227 | 0.0030 |
| bisnorabieta-8,11,13-triene | - | 0.0013 |
| *n*-hexadecanoic acid | - | 0.0738 |
| montanin-A | 0.101 | 0.0629 |

## ALKANES

***Table 1b.*** - Concentration (mg $g^{-1}$ of sample) of alkanes present in samples of galls and leaves of *Teucrium gnaphalodes* determined by GC-MS analysis of their $CH_2Cl_2$ extracts.

| ALKANE | Leaves | Galls |
|---|---|---|
| *n*-tricosane | 0.004 | 0.0034 |
| *n*-tetracosane | 0.008 | 0.0045 |
| *n*-pentacosane | 0.246 | 0.0237 |
| *n*-hexacosane | 0.021 | 0.0128 |
| *n*-heptacosane | 0.417 | 0.0820 |
| *n*-octacosane | 0.046 | 0.0216 |
| n-nonacosane | 0.558 | 0.1617 |
| *n*-triacontane | 0.060 | 0.0263 |
| *n*-hentriacontane | 0.570 | 0.1699 |
| *n*-dotriacontane | 0.062 | 0.0249 |
| *n*-tritriacontane | 0.404 | 0.1491 |
| *n*-tetratriacontane | 0.024 | 0.0142 |
| *n*-pentatriacontane | - | 0.0148 |

of the protein insect cuticle (Fig. 2). The EDS-ESEM chemical analyses of several ommatidia zones in the *Copium* specimen eyes display carbon contents ranging from 15 to 17%, phosphorous from 8 to 10%, oxygen ~62%, and calcium from 8.5 to 10.5%, assuming the existence of nondetected hydrogen element. Randomly, minor amounts of strontium up to 1%, magnesium up to 2%, and potassium up to 0.5% were also detected (Fig. 3). These noticeable amounts of 2% Mg and 1% Sr could be held in $Ca^{2+}$ structural positions in calcium-phosphate mineralizations, e.g., HAP, since these cations could be exchanged because they have similar-size atomic radii. Cuticle areas are mainly oxygen (72%), carbon (27%), and nondetectable hydrogen, with accessorial amounts of sodium, magnesium, aluminum, sulfur, potassium, and zinc chemical elements detected with the EDS probe. The molecular data on chemical bonds were recorded using a new Thermo Fisher DXR microscope to focus the Raman beam of few squared microns onto the red *Copium* eye ommatidia (Fig. 4). The most helpful region of the Raman spectrum ranges from 800 to 1700 $cm^{-1}$ since it attaches together Raman modes attributable to $PO_4$ and P-OH of phosphates, C-H and C-C of phospholipids, C-H of amide-S, helix of amide III, cytochrome P-450scc, etc. (Fig. 4). This Raman spectrum average is probably a mixture of an external protein cuticle, mineral calcium-phosphate phases, and a red fluid composed of phospholipids and bloody compounds, such as hemoglobin, erythrocytes, and cytochrome P-450scc. We here follow the Penel table[23] of Raman bands ($\Delta cm^{-1}$) and assignments for bone, collagen, lipids, and blood to perform mode assignations of the *Copium* eye

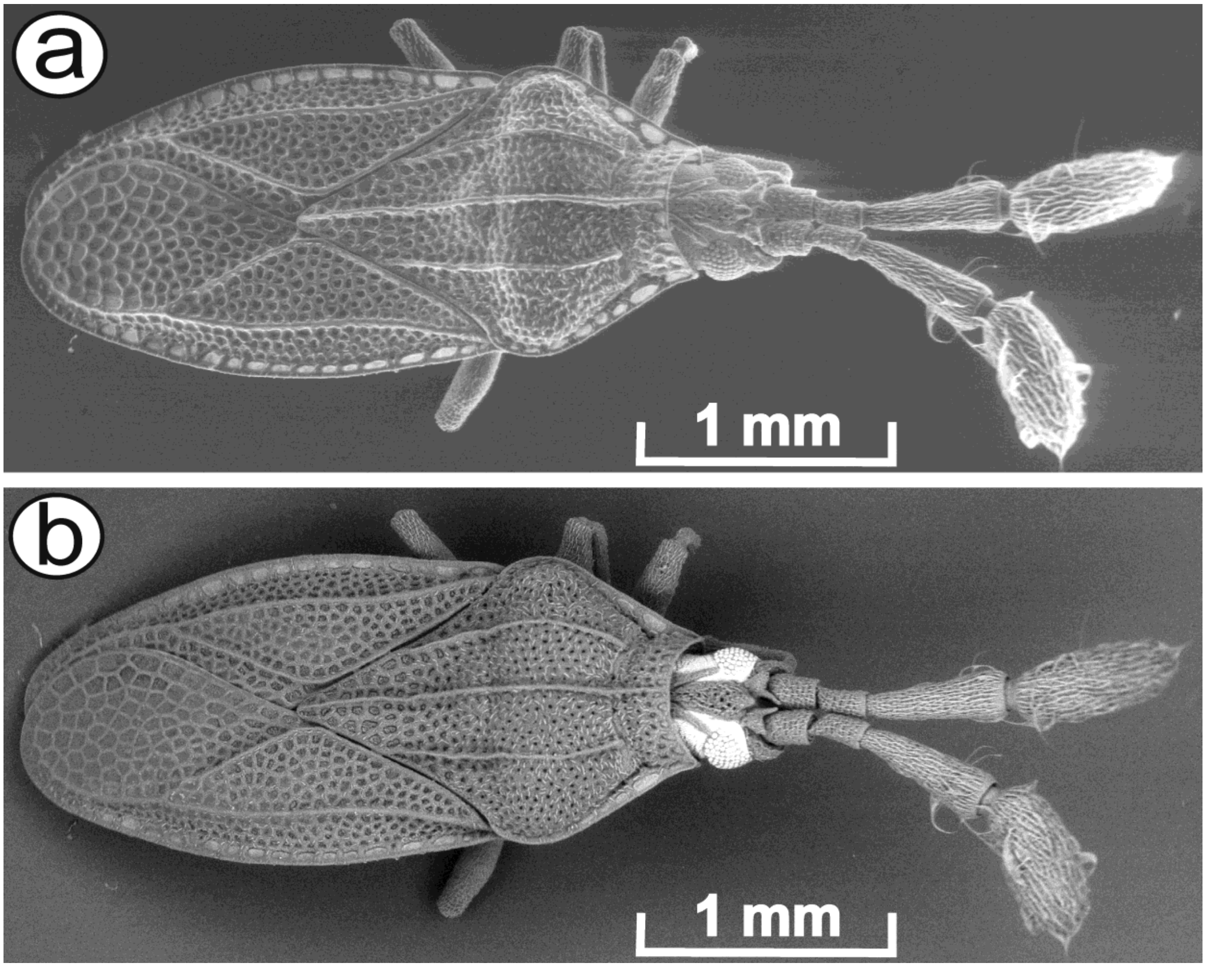

**FIGURE 2.** *Copium* adult specimen. (a) ESEM image of the whole *Copium* insect using the SED, displaying only topographic details. (b) The same ESEM image using the BSED, highlighting the eyes by their higher amount of Ca and P elements in comparison with the proteinaceous carbon areas.

spectrum, as follows: 850 $cm^{-1}$ δ(C-C-H) aromatic; 873 $cm^{-1}$ ν(C-C) of the hemoglobin variant L-isoleucine (IIe), valine, threonine, or tyrosine amino acids probably forming protein kinase-C since this kinase enzyme modifies other proteins by phosphorylation mechanisms adding chemically phosphate groups to them. Protein-kinase-c is required for light adaptation in insect photoreceptors[27]. The 920-$cm^{-1}$ Raman peak is linked with P-OH stretching and the ν(C-C) of hydroxylated proline. The prolyl-hydroxylase notably increases the structural stability of collagen. The hydroxylation of the proline amino acid is an important biochemical process for maintaining the connective tissue of higher organisms; 936 $cm^{-1}$ the nonaromatic n(C-C) mode of valine, lysine, or leucine; 960 $cm^{-1}$ nonaromatic mode ν1 $PO_4^{3-}$; 975 $cm^{-1}$ mode of amide III; 1130 $cm^{-1}$ of ν(C-C)trans of phospholipids and proteins; 1225 $cm^{-1}$ $\nu_{5+}$ $\nu_5$ modes of hemoglobin; 1266 $cm^{-1}$ mode of amide III; 1296 $cm^{-1}$ modes δ(=CH) of phospholipids; the band centered at 1340 $cm^{-1}$ is commonly assigned to protein α-helices of amide III; 1385 $cm^{-1}$, we attribute this band to amide-S ($C_\alpha$-H bending), however, the antisymmetric N–O stretching bands typical of free nitrate ions at 1385 $cm^{-1}$ and the band appearing at 1385 $cm^{-1}$, attributable to the νas-$SO_2$ mode of organic covalent sulfates, cannot be disregarded; 1485 $cm^{-1}$ $\nu_3$ mode cytochrome P-450scc; 1610 $cm^{-1}$ $\nu_{10}$ mode cytochrome P-450scc; 1530 $cm^{-1}$ (-C=C-) polyene erythrocyte carotenoid (Fig. 4). In addition, we also consider the described methodology for determining protein secondary structure, which assigns the 1267 $cm^{-1}$ band to amide III and the 1386 $cm^{-1}$ to the Cα–H amide bending vibrations[26]; this 1385 $cm^{-1}$ was

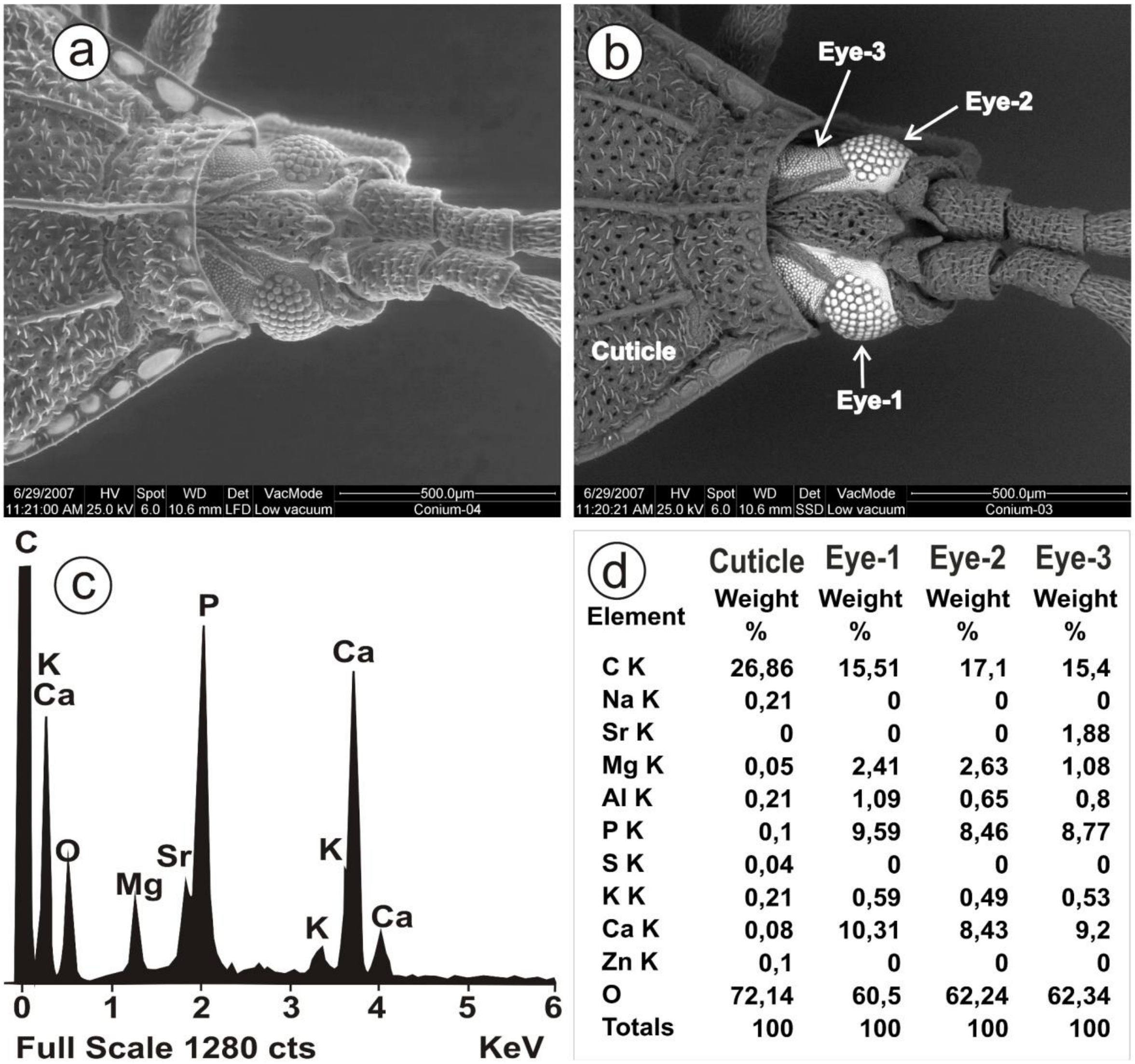

| Element | Cuticle Weight % | Eye-1 Weight % | Eye-2 Weight % | Eye-3 Weight % |
|---|---|---|---|---|
| C K | 26,86 | 15,51 | 17,1 | 15,4 |
| Na K | 0,21 | 0 | 0 | 0 |
| Sr K | 0 | 0 | 0 | 1,88 |
| Mg K | 0,05 | 2,41 | 2,63 | 1,08 |
| Al K | 0,21 | 1,09 | 0,65 | 0,8 |
| P K | 0,1 | 9,59 | 8,46 | 8,77 |
| S K | 0,04 | 0 | 0 | 0 |
| K K | 0,21 | 0,59 | 0,49 | 0,53 |
| Ca K | 0,08 | 10,31 | 8,43 | 9,2 |
| Zn K | 0,1 | 0 | 0 | 0 |
| O | 72,14 | 60,5 | 62,24 | 62,34 |
| Totals | 100 | 100 | 100 | 100 |

**FIGURE 3.** *Copium* eye ommatidium chemical EDS analyses. (a) Detail of the ESEM image under secondary electrons; (b) the same ESEM image by backscattering, including the spot analyses positions; (c) a representative EDS spectrum; (d) chemical spot EDS analyses taken on the ommatidia areas.

previously linked with amide-S, i.e., a nonhelicoidal structure as Cα–H bending mode[24] and finally, the attribution of 1485 $cm^{-1}$ and 1620 $cm^{-1}$ bands to the modes ν10 and ν3 of the oxidized cytochrome P-450scc[25].

Despite the molecular information inferred from the Raman assignation bands, additional details were obtained under the FESEM microscope since it has top enlargement capabilities. The intercomparison between both *Copium* ommatidia images (Fig. 5a SEM-BSED and Fig. 5b FESEM) reveals the existence of epithelial tissue outer onto the ommatidia surface. These squeezed tissue folds were formed by dehydration in the FESEM chamber at high vacuum since the tissue was previously stored in ethanol. This detail cannot be observed at lower magnification in the ESEM microscope. This external ommatidium membrane possibly is made of phospholipidic cholesterol or lipophilic molecules, including farnesyl and geranyl diphosphates[28]. We also employed spatially resolved luminescence techniques, such as CL and CLSM images of the *Copium* ommatidia eyes, to learn additional details of the calcium-

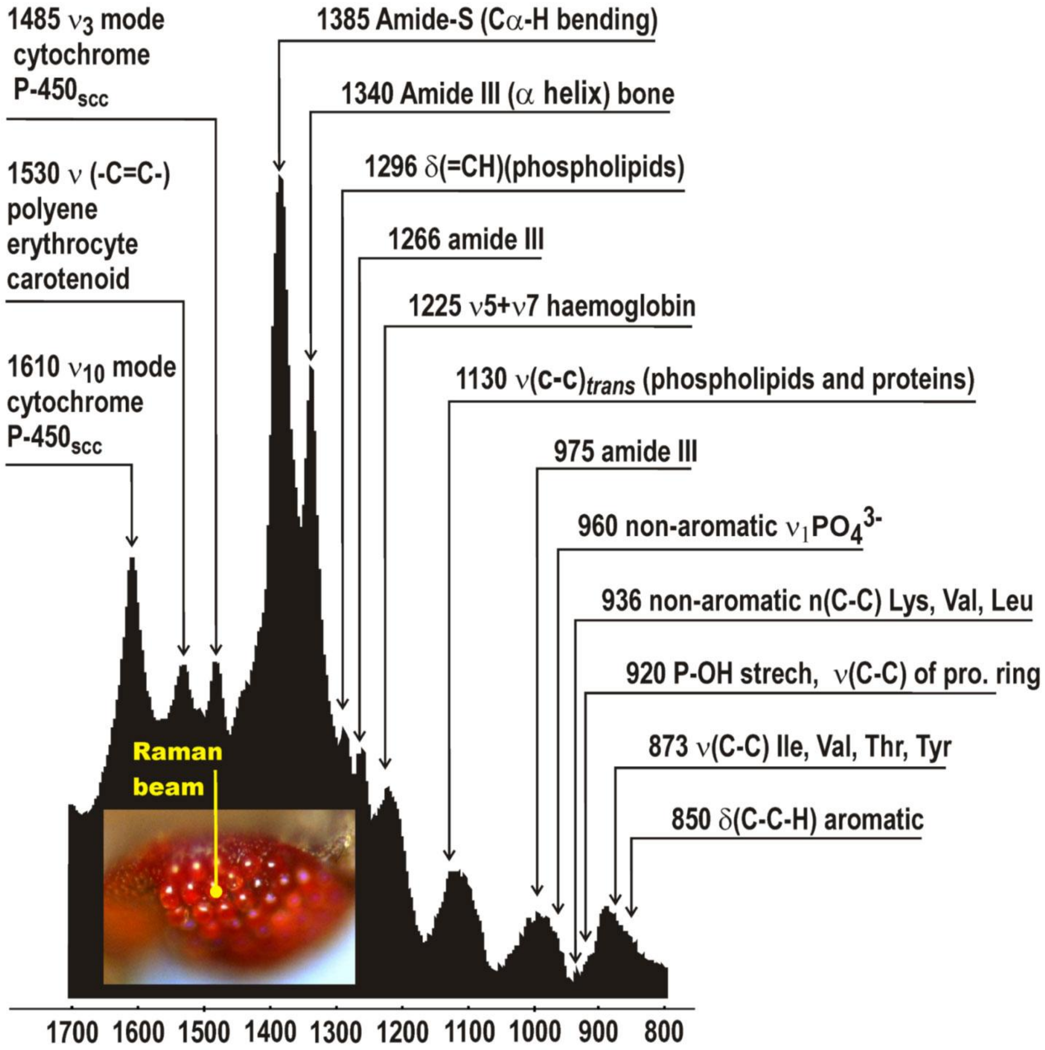

**FIGURE 4**. *Copium* eye ommatidium representative Raman spectrum, including Raman bands ($\Delta cm^{-1}$) and assignments following the table of Penel et al.[23] for bone, collagen, lipids, and blood to perform node assignations.

phosphate mineralization. Fig. 5c depicts a CL panchromatic image recorded by the monoCL3 probe coupled to our SEM FEI microscope; the whole area looks white under the backscattering SEM, which must be interpreted as a mineralized area of calcium phosphate; furthermore, switching to the CL panchromatic image, the four *Copium* setae turn bright white in color, while the four ommatidia exhibit gray color. This clear contrast among CL intensities of different elements, i.e., ommatidia and setae, of the CL image (Fig. 5c) demonstrates relative differential CL intensities, probably explained by a more mineralized hard structure, e.g., more HAP, in comparison with the lesser amount of mineralization of the ommatidia background as inferred from its small CL emission. The CL images of the setae show also an external peripheral collagen cuticle. Fig. 5d is a photoluminescent image of the *Copium* ommatidia taken with the CLSM microscope during several recording series. It is interesting to observe as the calcium-phosphate mineralization develops simultaneously in two different ways: (1) such as isolated groups of crystals into the ommatidia cores and (2) implanted in the internal side wall of the ommatidium. In both

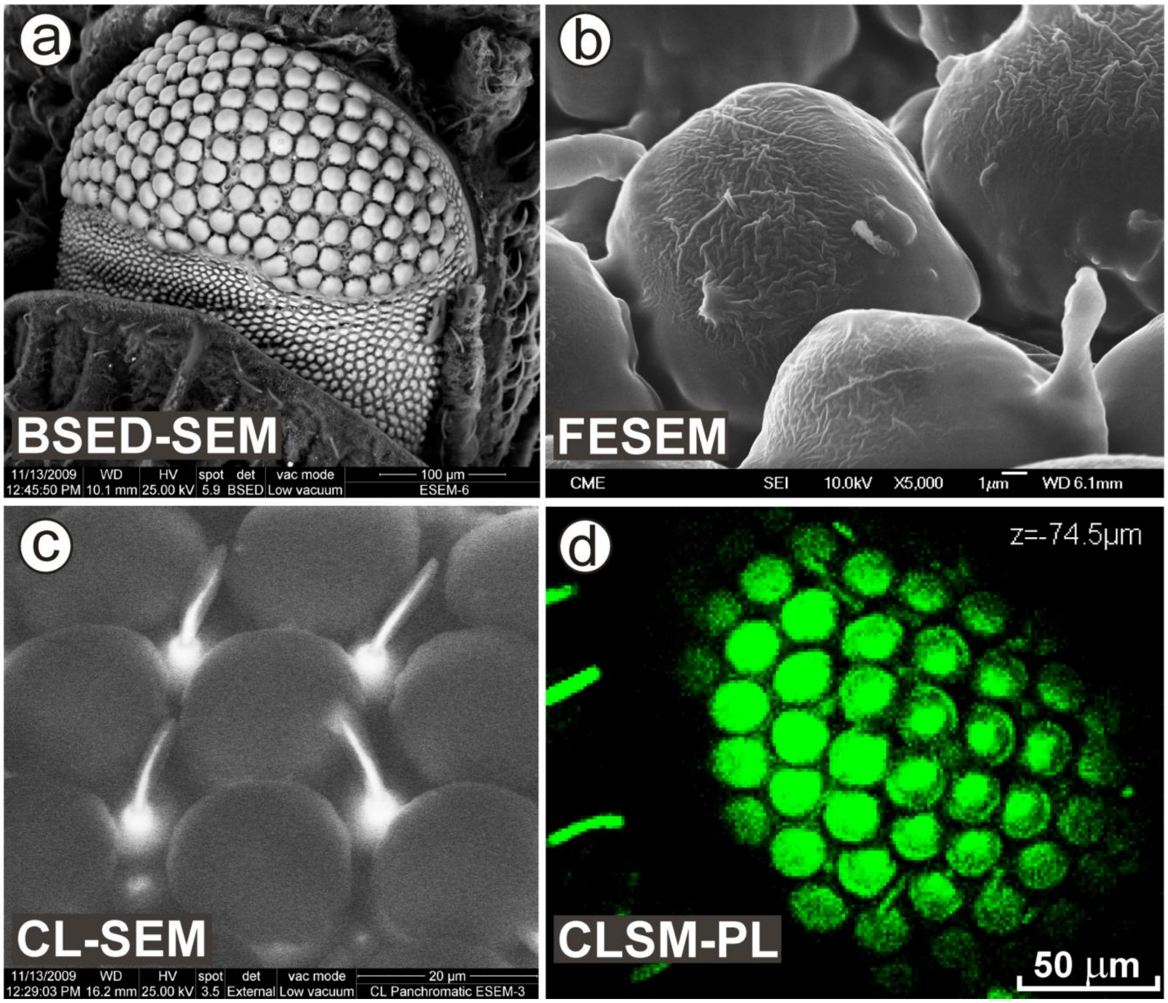

**FIGURE 5.** (a) Backscattering image under SEM of the calcium-phosphate mineralized eye showing some setae among the *Copium*. (b) FESEM image detailing the squeezed external proteinaceous-collagen membrane by dehydration. (c) CL panchromatic image under ESEM showing the most intense CL emission from setae in comparison with the surrounding ommatidia. (d) Photoluminescence image recorded in the CLSM, depicting both the mineralized areas as core crystal aggregates and on the internal ommatidium membrane walls.

cases, the calcium-phosphate crystals are formed according to the crystal growth theory by which crystals grow from specific former nuclei, or impurities, and from supporting surfaces by epitaxial mechanisms. Fig. 6 joins several spectra CL of bond materials, including *Copium* eye seta, ommatidium, and cuticle, together with other comparative patterns of chemical collagen, medieval human femur, Siberian mammoth ivory, human liver cysteine calculi, etc. All these CL spectra show the common feature of their similar curve shapes matching the chemical pure collagen; this plot shows as the collagen materials are much more cathodoluminescent than the calcium-phosphate phases, including HAP. The comparison between the spectra CL curves confirm that the *Copium* eye setae material is more luminescent than the ommatidium background. These surprising CL curves of organic materials were recorded with a new monoCL3 detector of the Gatam Company, which has up to a 75% light recording efficiency. Fig. 6 confirms the collagen composition of the *Copium* ommatidia with a close shape of CL curve similar to other collagen HAP complexes used as CL patterns. The *Copium* ommatidia responds to both hormones and terpenic environment, regulating the concentration of ions within the extracellular matrix and secreting

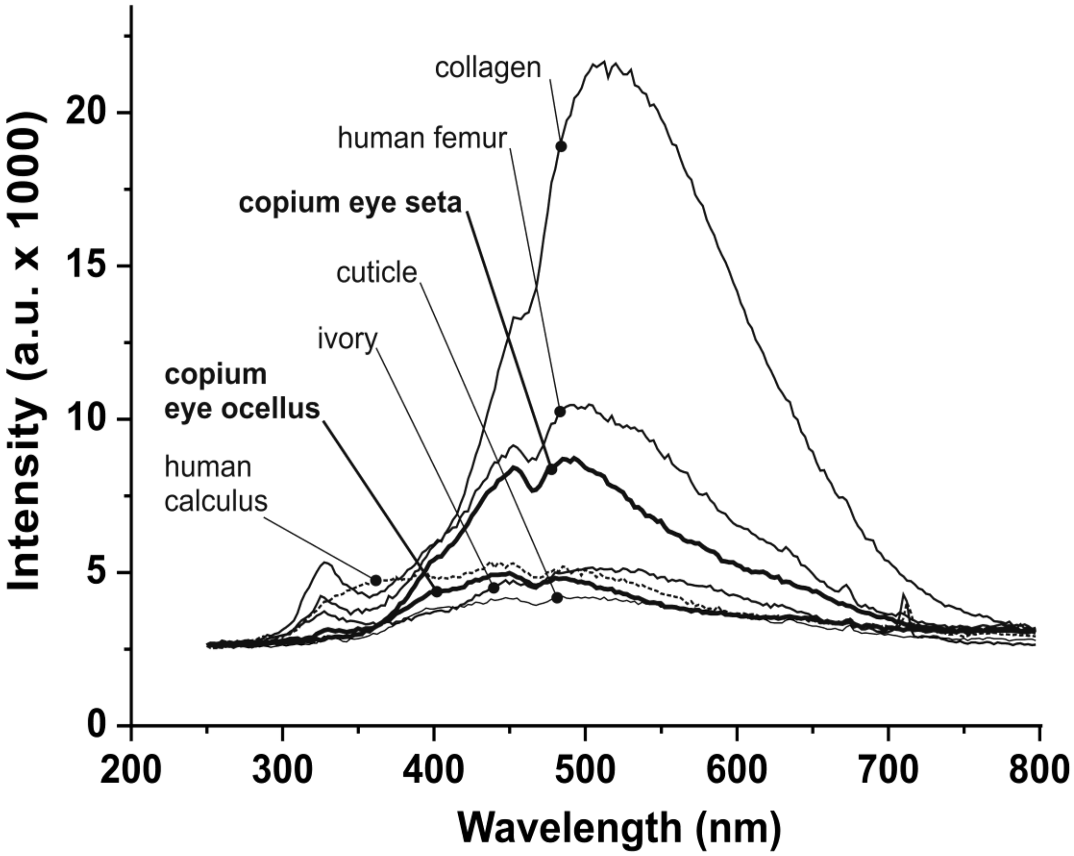

**FIGURE 6.** Spectra CL curves of collagen-calcium-phosphate bond materials, including *Copium* eye seta, ommatidium, and cuticle, together with other comparative patterns of chemical collagen, medieval human femur, Siberian mammoth ivory, and human calculus.

macromolecules whose properties determine the ability of the matrix to be calcified. The mitochondria within the cells accumulate calcium and phosphate, releasing these ions into the matrix as calcification progresses. Among the macromolecules secreted by the cell, collagen provides the support for the HAP crystals[29]; proteoglycans serve to control the extent and/or progress of mineralization. The proteoglycans, glycoproteins, enzymes, and the collagen itself in ommatidia cells determine the nature of the matrix, while phosphoproteins, proteolipids, and phospholipids may serve as HAP nucleators or as surfaces upon which the calcium phosphate is deposited[30]. The light-induced lowering of calcium levels, probably also associated with the calcium-phosphate crystallization, aids the recovery of excited neurons to a passive, “dark” state; later, the cycle starts again upon detection of light. Although ommochromes are responsible for the colors of insect eyes, they are not known to be involved directly in photoreception. A comparative study[7] on the red-eyed (wild) and white-eyed (mutant) *Drosophila melanogaster*, and the black (wild) and red (mutant) eyes present in *Triatoma infestans*, shows that the eye color differences in *T. infestans Klug* (Hemiptera, Reduviidae) appear associated with the xanthommatin concentration, with a smaller content of ommochrome in red eyes. In our case, the *Teucrium* gall seems to be an exceptional micromodel of a closed biological microenvironment to facilitate the insect growth, including extracellular vesicles with calcium-phosphate deposits working as real ommatidia eyes.

## CONCLUSIONS

*Copium teucrii* insects induce histological hyperplasia in *Teucrium gnaphalodes* plants, forming vegetable galls in which they live and grow. Adult *Copium* specimens leaving the gall develop red eyes with a calcium-phosphate mineralization easily detectable under ESEM-EDS, SEM-CL, and CLSM. The calcium-phosphate mineralization is formed and constrained to the *Copium* ommatidia, which are both matrix vesicles generating mixtures of apatite-collagen and operational compound eyes of the insect. *Copium* also shows setae (bristles) that project through their ommatidia made by a most hard-mineralized calcium phosphate as it was observed by panchromatic ESEM-CL mapping. Micro-Raman spectra taken in a *Copium* ommatidium exhibit characteristic bands attributable to bone tissue apatite biomaterials, such as bone, collagen, lipids, and blood, i.e., peptides, amide-S, amide-II, amide-III, and cytochrome P-450scc. *Copium* ommatidia are membrane-bound matrix vesicles concentrating calcium phosphate and cytochrome phases, lipids, collagen, opsin protein, and other undetermined compounds. Microscopes are different and complementary: The ESEM-BSED detector outlines the calcium-phosphate areas (ommatidia); FESEM displays the ommatidium external membrane; ESEM-EDS analyzes the chemical composition of C, P, Ca, and accessorial Sr and Mg; the ESEM-CL panchromatic imaging allows differentiating among calcium-phosphate ommatidium and calcium-phosphate setae, which are more mineralized. The CLSM microscope depicts the internal distribution of calcium phosphate along with internal membrane sides and ommatidia nuclei, and micro-Raman depicts the molecular information on specific ommatidia. *Teucrium* galls provide a microenvironment in which the presence of terpene compounds and low-volatility *n*-alkanes could help in *Copium* development. The unusual calcium-phosphate mineralization structures occurring in *Copium* eyes were found in *T. gnaphalodes* growing in calcium-rich limestone and gypsum. However, further studies are required to throw some light on both the factors responsible for these surprising eye characteristics and on their relevance in *Copium* ecology.

## ACKNOWLEDGMENTS

We are grateful to projects CGL2009-09247 and CGL2009-10111 of the Spanish PLAN NACIONAL DE I+D+i for financial support.
